# Production and quality of clinical practice guidelines in Argentina (1994–2004): a cross-sectional study

**DOI:** 10.1186/1748-5908-3-43

**Published:** 2008-10-13

**Authors:** María Eugenia Esandi, Zulma Ortiz, Evelina Chapman, Marcelo García Dieguez, Raúl Mejía, Ricardo Bernztein

**Affiliations:** 1 Instituto de Investigaciones Epidemiológicas, Academia Nacional de Medicina, Ciudad Autónoma de Buenos Aires, Argentina; 2 Area de Estudio de los determinantes epidemiológicos de la salud, Universidad Nacional del Sur, Bahía Blanca, Argentina; 3 Programa de Medicina Interna General, Hospital de Clínicas, Universidad de Buenos Aires, Argentina; 4 Hospital de Pediatría "Prof. Dr. Juan P. Garrahan", Buenos Aires, Argentina

## Abstract

**Background:**

In the last decades, a sustained increment of Clinical Practice Guidelines (CPG) production in the world has been accompanied by a growing concern about their quality. Many studies related to quality assessment of guidelines produced in High Income Countries were published; however, evidence on this topic is scarce in Low and Middle Income Countries (LMIC). The objectives of this research were: a) to describe guideline production in Argentina at different levels of the health system (macro, meso and micro) from 1994 to 2004; and b) to assess their quality by using the AGREE instrument.

**Methods:**

A cross-sectional study was undertaken to describe guidelines production in Argentina between 1994 and 2004. CPG were identified through Internet and electronic databases (MEDLINE and LILACS). Explicit inclusion and exclusion criteria were used to select guidelines. Each CPG was independently assessed by two reviewers using the AGREE instrument. Domain scores were calculated as recommended by the AGREE Collaboration. The internal consistency of each domain was evaluated using Cronbach's alpha and inter-observer agreement by the Intraclass Correlation Coefficient (ICC).

**Results:**

A total amount of 431 potential CPG were identified, but only 144 were considered CPG. At the end, 101 CPG were included for further assessment. Median standardized score for each domain were: scope = 39%; stakeholder involvement = 13%; rigour of development = 10%; clarity = 42%; applicability = 6%; editorial independence = 0%. Only 22 CPG were recommended with modifications by both appraisers. ICC and Cronbach's alpha for each domain were in all cases moderate or high (greater than 0.40), except for editorial independence.

**Conclusion:**

This study has systematically employed the AGREE instrument for the critical assessment of guidelines produced in a LMIC. Guideline development and diffusion in Argentina from 1994 to 2004 shows a constant increment, although quality of reporting did not improve; moreover, in some aspects it seemed to decline. Much room for improvement of the guideline development process was found at all levels of the health system.

## Background

Clinical Practice Guidelines (CPG) are one of the tools most frequently used by health professionals to improve the micro level decision-making process. As defined by the Institute of Medicine (IOM), they are "systematically developed statements to assist practitioner and patient decisions about appropriate health care for specific clinical circumstances"[1]. Guidelines may offer concise instructions on which diagnostic or screening tests need to be order, how to provide medical or surgical services, how long patients should stay in hospital, or other details of clinical practice [2]. The ultimate purpose of developing and using guidelines is to improve the quality of care provided, particularly in areas of clinical uncertainty.

In the last years, a sustained increment in guidelines production was observed all over the world, especially in United States, Canada, Australia, New Zealand and European countries. Most of these countries have developed national programs for CPG production, dissemination and implementation in order to increase the effectiveness and quality of the health system [3].

Some of these initiatives, which were originally conceived as individual efforts, have been strongly improved by international cooperation through organizations such as the Guidelines International Network (GIN) [4] and the Appraisal of Guidelines for Research and Evaluation (AGREE) Research Trust [5]. The need for harmonizing and systematizing guideline development and assessment was one of the most important reasons that prompted the establishment of these international organizations [6].

A "good quality guideline" is that one that ultimately leads to improve patient outcome. However, quality of guideline is indirectly measured by assessing in what degree guideline producers minimized potential biases that could occur in the development process and affect validity of its recommendations [7]. Wrong recommendations affect health professionals' credibility on guidelines, and consequently, limit their adoption [2].

In 1999, Shaneyfelt and col. assessed quality of CPG published in Medline between 1985 and 1997 by using a systematically developed instrument. The majority of 279 assessed guidelines did not meet the pre-established methodological standards, being rigour of recommendations one of the most deficiently reported [8]. Similar results were reported by Cluzeau and col.[9], Grilli and col.[10] and Graham and col.[11] in 1999, 2000 y 2001, respectively. In 2003, the AGREE collaboration (currently the AGREE Research Trust) published the results of the first international project aimed at developing and validating a generic instrument for guidelines assessment [7]. This instrument has been translated to different languages, extending its use throughout the world. In the recent years, several studies showed methodology deficiencies in guideline development by using the AGREE instrument [12-14].

Almost all research about quality assessment of CPG has been undertaken in High Income Countries (HIC). Studies about quality of guidelines produced and diffused in Low and Middle Income Countries (LMIC), and particularly in Latin America, are scarce [15]. In Argentina, although many different institutions are interested in CPG development, there is no information about quantity of guidelines produced, and moreover, quality of these documents. The purpose of this research is to describe trends in guidelines production in Argentina and to assess their quality by using the AGREE instrument.

## Methods

A cross-sectional study was undertaken to describe guidelines production in Argentina between 1994 and 2004. Documents were considered as CPG if: 1) they included explicit recommendations targeted to health professional decision-making, being this related to: screening and primary prevention, diagnosis, treatment and secondary prevention and/or rehabilitation; 2) they contained bibliographic references and in the case of consensus, participants or responsible institutions were described; 3) they were produced and diffused in the period of study (January 1994- December 2004) and could be freely accessed. The exclusion criteria were: 1) guidelines targeted to patients (patients'guidelines) and/or exclusively oriented to health services organization and not to clinical decision-making; 2) guidelines for which it was not possible to determine if a systematic process was applied in their development (i.e. documents that lacked an explanation of the guideline development methodology that had been used; documents diffused as brief reports which only contained a set of recommendations; documents referred to as guidelines, but that were undertaken by only one author without any reference to the methodology applied); 3) guidelines whose year of development could not be established as it was not stated; 4) guidelines that were not produced by an Argentine institution (adapted guidelines were included only when the adaptation process was explicitly explained).

Electronic databases searching (EDS): executed by an expert by means of the strategy described in Table 1. It was initially developed to be performed in MEDLINE under PubMed platform. Afterwards it was adapted to be used in regional databases (LILACS). All retrieved articles were assessed by the principal investigator. An Internet searching (IS) was also perfomed to identify CPG posted on Websites. Subsequent institutions were classified according to the level of the health system to which they belong (macro, meso and micro level). Table 2 shows the definition used to describe each level.

**Table 1:** Description of the searching strategy employed in Medline

**N° Step**	**Description of the Search Strategy**
1	("guideline" [Publication Type] OR "guidelines" [MeSH Terms] OR "guidelines" [Text Word])
2	("consensus" [MeSH Terms] OR consensus [Text Word])
3	algoryth*
4	#1 OR #2 OR #3
5	argentin*
6	#4 AND #5

**Table 2:** Internet searching: institutions included at each level of Health System

**Level of the Health System**	**Definition**
**Macro Level**	Organisms of the national, provincial and municipal State in charge of health policy formulation, execution and control. It includes: a) **National State**: Ministry of Health, including all its decentralized departments, secretaries'offices and their dependent organizations that have health promotion, prevention and care as one of their specific goals; b) **Provincial State**: Health ministries or offices of the provincial government; c) **Municipal State**: Health offices of cities that were provincial capitals or have more than 250.000 inhabitants.
	Websites of Health Technology Assessment agencies were also included at this level.
**Meso Level**	Intermediate institutions of the public, private and social security sector that provide or manage health services. It includes: individual providers, organizations of providers and health assurance institutions.
**Micro Level**	It is theoretically constituted by individual health professionals. In practice, scientific or professional associations were selected. Only national organizations were included.

Quality guideline assessment was perfomed through the AGREE instrument. This instrument was selected amongst others as it is the only one that covers practically all the relevant dimensions of the guideline development process; it has been internationally validated; it has fewer items and uses a numerical scale that facilitates the analysis and comparison of the results [7,16,17]. This instrument has been widely used all over the world, mainly as a result of its translation into many other languages, including Spanish. This version was already tested in Spain and it proved to be reliable and feasible to apply [13].

A total of 30 health professionals distributed throughout the country were invited to participate in the assessment phase. To be considered elegible, professionals should have had at least one of the following criteria: a) previous clinical epidemiology background; b) knowledge on guidelines development; c) experience with the AGREE instrument. Those professionals that accepted the invitation and fulfilled the eligibility criteria were trained in the use of the AGREE instrument. A 45 days-e-learning program was developed in three stages: I. Self-reading of the tool-kit (15 days): all participants were provided with the Spanish version of the AGREE instrument, the Spanish and English version of the Training Manual. II. Pilot CPG critical appraisal (15 days): one CPG was assessed independently by all professionals. A data collection form designed on an Excel sheet, accompanied by a user-guide were sent to each participant. Results of assessments were returned to the researcher team by e-mail. Results were compared and divergences were discussed with each appraiser through an individual feedback. III. Adjustment phase (15 days): during this last stage, unresolved doubts could be raised by each participant in order to be discussed with the researcher team. Only those professionals that suscessfully completed this three-stage training were formally accepted as appraisers (n = 23). No one received any honorarium.

Whenever was possible, guidelines were assigned taking into account the expertise and specialty of each appraiser. The median numbers of guidelines assessed by each appraiser was 8. According to the AGREE collaboration the domain scores of each CPG were individually considered and scores of individual items in each domain were summed and standardized as a percentage of the maximum possible score for that domain, taking into account the number of appraisers. Relation between quality domain scores and other variables (year of production, level of the health system, guideline publication and category) was assessed through bivariate analysis. As distribution of the dependent variable was generally asymetrical, non- parametrics tests were used. The Kruskal-Wallis test was used to test the statistical significance of the difference when categories of the grouping variable was higher than 2. When categories were only 2, the Mann Whitney Test was applied.

The internal consistency of each domain was evaluated using Cronbach's alpha. The reliability between appraisers was determined for each question and each domain of the AGREE. Intraclass correlation coefficients (ICC) were calculated within each pair of appraisers and across the pool of appraisers. ICCs and kappa values above 0.75 were considered to represent good, 0.40–0.75 moderate and <0.40 poor reliability.

Feasibility of the instrument was assessed through an *ad-hoc *instrument that contained two dimensions: usefulness and simplicity; both dimensions were assessed through a 1–5 scale, being 5 the highest score.

## Results

### Guidelines production in Argentina

A total amount of 431 documents were identified through the combination of both searching strategies (EDS and IS) – websites of 247 institutions were assessed through IS-. Most of retrieved documents 84% (363) were identified through IS and 16% through EDS. Of the 431 documents, 33% (144) fulfilled the inclusion criteria. Excluded documents were classified as follows: i) lacking of year of publication or diffusion (n = 121), lacking of references or authors' identification in the case of consensus (n = 110), lacking of a methodology section (n = 107); ii) document oriented to health services organization, targeted to macro level decisions for coverage purpose and/or exclusively targeted to patients (n = 103); iii) not free access document (n = 54); iv) documents that only contains a set of recommendations or were not developed in the period of study (n = 12). A number of documents accomplished more than one exclusion criteria.

Of the 144 CPG selected in 7 cases the full-text could not be retrieved. We intended to assess the remaining 137 CPG by two appraisers. Due to non response, 28 CPG were assessed by one appraiser and 8 CPG by no appraiser. These CPG were excluded from our analysis. We present the results of the 101 CPGs that were assessed by two appraisers.

**Figure 1:**
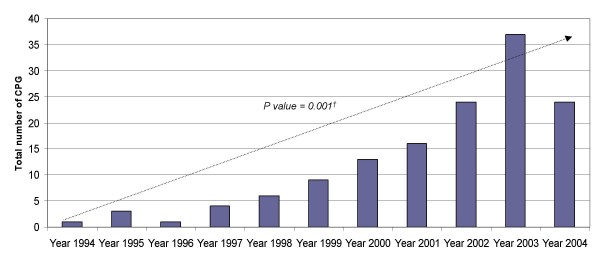
Temporal evolution of the total number of CPG produced per year *Ref: (†): Estimated by χ^2 ^for trends*.

CPG production increased along the study period, being this positive trend statistically significant (Figure 1). Scientific societies were the principal CPG producer during the study period. Interaction of institutions belonging to different levels of the health system was unfrequent (12% – 17/144). The type of interaction most frequently observed was between the macro level (the National MOH) and scientific societies (Table 3). Most CPG were about diagnosis and treatment/management: only 22% (31/144) were oriented to prevention and screening practices. Although international CPG were referenced in some of the selected CPG, an explicit adaptation process was never reported.

**Table 3:** Amount of CPG produced by level and institutions of the Health System

**Health System Level**	**Type of Institution**	**Total number of CPG**
Macro	National Health Ministry	13
	Provincial Public Health Department	0
	Local Public Health Department	0
	Health Technology Assessment Agencies (n = 2)	0
Meso	Public and Private Hospitals	15
	Hospitals Network	0
	Prepaid Enterprises	0
	Social Security Organizations	23
Micro	Scientific Societies	76
More than one level	Macro/Meso/Micro	3
	Macro/Micro	14
	**TOTAL**	**144**

### Quality assessment

Of the 144 CPG selected, 101 (71%) were assessed and 43 were eliminated (in 7 cases the full-text could not be retrieved; 8 CPG could not be assessed by none appraisers and 28 were appraised by only one of the two appraisers).

**Figure 2:**
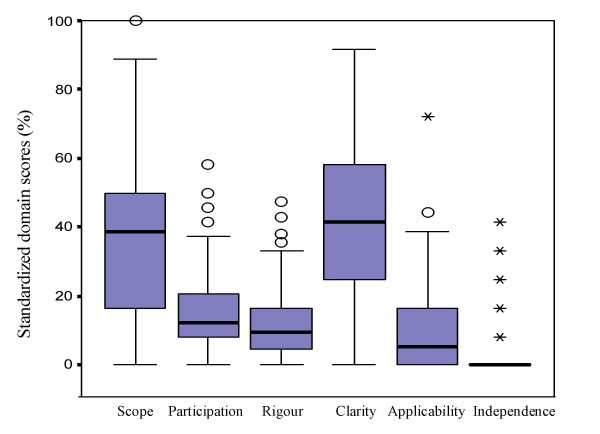
Results of the analysis of the 101 Argentine guidelines on the six AGREE instrument domains.

The majority of the CPG assessed received very low scores in nearly all domains (Figure 2). Over 80% of the CPG were assessed with scores lower than 50%, except in those domains corresponding to "clarity" and "scope". In comparison to the results of the other domains, clarity was the best scored aspect of CPG. Analysis by item showed median values lower than 3 in the 23 items of the AGREE instrument: 14 items received the lowest possible score (1) (Table 4).

**Table 4:** Scores by item of the AGREE instrument

**Domains and items of the AGREE instrument**	Median value	Interquartile range
**Domain 1: Scope and purpose**		
1. The overall objective(s) of the guideline is (are) specifically described	1.5	1.5
2. The clinical question(s) covered by the guideline is (are) specifically described	2	1
3. The patients to whom the guideline is meant to apply are specifically described	2.5	1.5
**Domain 2: Stakeholder involvement**		
4. The guideline development group includes individuals from all the relevant professional groups	1.5	1
5. The patients' views and preferences have been sought	1	0.5
6. The target users of the guideline are clearly defined	1	1
7. The guideline has been piloted among end users	1	0
**Domain 3: Rigour of development**		
8. The systematic methods were used to search for evidence	1	0
9. The criteria for selecting the evidence are clearly described	1	0
10. The methods used for formulating the recommendations are clearly described	1.5	0.5
11. The health benefits, side effects and risks have been considered in formulating the recommendations	2	1
12. There is an explicit link between the recommendations and the supporting evidence	1	1
13. The guideline has been externally reviewed by experts prior to its publication	1	0.5
14. A procedure for updating the guideline is provided	1	0
**Domain 4: Clarity and presentation**		
15. The recommendations are specific and unambiguous	2.5	1.5
16. The different options for management of the condition are clearly presented	2.5	1.5
17. Key recommendations are easily identifiable	2.5	1.5
18. The guideline is supported with tools for application	1	0.5
**Domain 5: Applicability**		
19. The potential organizational barriers in applying the recommendations have been discussed	1	0.5
20. The potential cost implications of applying the recommendations have been considered	1	0
21. The guideline presents key review criteria for monitoring and/or audit purposes	1	1
**Domain 6: Editorial independence**		
22. The guideline is editorially independent from the funding body	1	0
23. Conflicts of interest of guideline development members have been recorded	1	0

**Figure 3:**
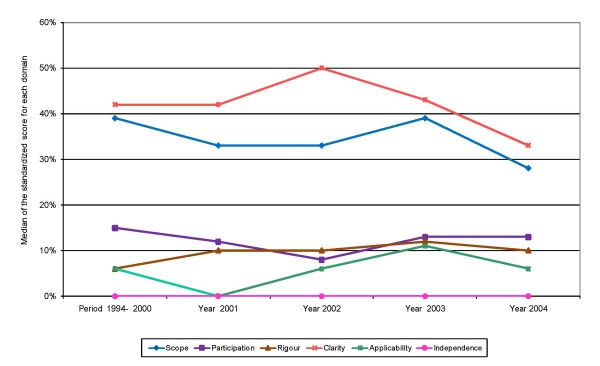
Temporal evolution of the median standardized score for each AGREE instrument domain.

There was no statistically significant difference in the median of standardized domain scores alongside the study period (Figure 3). No association between CPG quality and other variables, like method of diffusion of the guideline (published vs. not published), level of production (macro, meso, micro and interaction among levels), type of guideline (prevention vs. treatment/diagnosis management guideline) and scope (national vs. regional/local guidelines) was observed. Statistically significant differences were only observed among scores corresponding to the participation domain (guidelines produced by an interaction of institutions belonging to more than one level of the health system had higher scores than guidelines produced by institutions belonging to only one level) and clarity domain (prevention CPG had higher scores than treatment/diagnosis management guidelines) (Table 5).

**Table 5:** Comparison of CPG quality according to independent variables

	**Median [Interquartile range] of standardized domain scores**					
	**Scope**	**Participation**	**Rigour**	**Clarity**	**Applicability**	**Independence**
**Diffusion year**						
Period 1994–2000 (n = 22)	39% [40%]	15% [22%]	6% [10%]	42% [46%]	6% [17%]	0% [0%]
Year 2001 (n = 13)	33% [47%]	12% [29%]	10% [15%]	42% [46%]	0% [19%]	0% [0%]
Year 2002 (n = 19)	33% [28%]	8% [17%]	10% [15%]	50% [67%]	6% [6%]	0% [8%]
Year 2003 (n = 28)	39% [29%]	13% [13%]	12% [11%]	43% [40%]	11% [19%]	0% [0%]
Year 2004 (n = 19)	28% [39%]	13% [17%]	8% [14%]	33% [25%]	6% [11%]	0% [8%]
P value	*0,66*	*0,89*	*0,28*	*0,68*	*0,52*	*0,72*
**Publication**						
Published (n = 39)	39% [33%]	13% [46%]	10% [14%]	42% [29%]	6% [17%]	0% [0%]
Not Published (n = 62)	39% [33%]	10% [13%]	12% [14%]	44% [38%]	11% [17%]	0% [0%]
P value	*0,93*	*0.76*	*0,23*	*0,40*	*0,24*	*0,43*
**Level of the Health System**						
Macro (n = 12)	42% [44%]	**13% [15%]**	11% [13%]	61% [42%]	11% [18%]	0% [0%]
Meso (n = 25)	28% [31%]	**8% [13%]**	12% [12%]	37% [37%]	6% [11%]	0% [0%]
Micro (n = 49)	39% [32%]	**13% [17%]**	7% [14%]	41% [28%]	6% [11%]	0% [0%]
More than one level (n = 15)	39% [50%]	**21% [13%]**	12% [17%]	41% [37%]	6% [22%]	0% [0%]
P value	*0,12*	***0,01***	*0,24*	*0,11*	*0,21*	*0,94*
**Guideline Scope**						
National CPG (n = 74)	39% [33%]	13% [13%]	10% [15%]	42% [34%]	6% [18%]	0% [0%]
Regional/Local CPG (n = 27)	28% [25%]	8 % [13%]	12% [14%]	38% [38%]	6% [11% ]	0% [0%]
P value	*0.11*	*0.12*	*0.24*	*0.52*	*0.35*	*0.4*
**CPG category**						
Dx/treatment management (n = 71)	39% [28%]	13% [13%]	9% [14%]	**42% [33%]**	6% [17%]	0% [0%]
Prevention (n = 25)	39% [50%]	13% [19%]	9% [12%]	**54% [35%]**	6% [17%]	0% [0%]
P value	*0,19*	*0,33*	*0,53*	***0,02***	*0,85*	*0,77*

ICC and Cronbach's alpha for each domain were in all cases moderate or high (0.46–0.74), except for Editorial Independence which showed very low values (0.20) (Table 6). Most of appraisers considered that the AGREE instrument was useful and simple to apply (usefulness, median value: 5 and simplicity, median value: 4). Scope was selected by the group of appraisers as the most difficult domain to be assessed. Average time (standard deviation) employed per CPG assessed was 58 (± 36) minutes.

**Table 6:** Reliability scores of the AGREE instrument

	Reliability measures		
	Single Rater ICC (95% CI)	Average of raters ICC (95% CI)	Cronbach alpha
Scope	0,33 (0,24–0,42)	0,74 (0,65–0,81)	0.75
Participation	0,09 (0,05–0,16)	0,46 (0,28–0,61)	0.55
Rigour	0,09 (0,05–0,14)	0,58 (0,45–0,70)	0.67
Clarity	0,26 (0,19–0,34)	0,74 (0,65–0,81)	0.82
Applicability	0,16 (0,09–0,24)	0,53 (0,38–0,66)	0.56
Editorial Independence	0,05 (-0,02–0,16)	0,20 (-0,09–0,42)	0.23

## Discussion

In the last years, development of guidelines in Argentina has progressively increased; however, quality did not improve. This situation could be clearly resumed in the phrase of Sudlow and Thomson: "*Quantity but no quality*" [18].

Similiar results were reported by some studies performed in HIC between 1999 and 2005 [8-14,19]. In many cases, these findings could have contributed to prompt the establishement of national guidelines programs with the aim of systematizing the guideline development process. However, and even when, comparatively to LMIC, important improvements have been achieved in relation to guidelines production, this issue of "pluralism and low quality" still raises serious concerns in HIC. A recent report from the National Institute of Medicine (USA) questionated the validity and reliability of many guidelines produced in this country due to the lack of rigourness, objectivity and transparency of the development methodology that had been applied [20].

Quality of guidelines produced in LMIC, and particularly in Latin America, is practically unknown. To our knowledge, there is only one CPG quality assessment that precedes the Argentine research, which was performed in Brazil. In this study, twenty-eight guidelines developed by the Brazilian Medical Association were independently assessed by 2 appraisers using the AGREE instrument [15]. This is the second guideline appraisal study in a Latin American country, but on a larger scale than the first conducted in Brazil.

Quality of the assessed Argentine guidelines was far from ideal: scores were low and very low in all domains and items of the AGREE instrument. Many factors might have contributed to this situation.

First, low quality could have been the result of the absence of an explicit policy for guidelines production and evaluation during the period under assessment. Argentine health system is highly complex and integration of activities of multiple stakeholders is difficult to achieve without a clear guidance. Although many institutions of the three levels of the health system participated in this process, a more integrated approach is required in order to balance the interests, preferences and knowledge of different stakeholders whose participation in the guideline development process is not only required, but need to be guaranteed.

Second, low quality scores of Argentine guidelines could be explained by a slower penetration and consolidation of the evidence-based movement in LMIC countries in comparison to developed countries. As described by Burgers, development of guidelines in Europe, Australia and North America started in the 80's and 90's [3]. In the United States, the Consensus Development Program at the National Institute of Health developed its first guideline in 1977. In the last 30 years, all these organizations have accumulated a vast experience in guideline development, dissemination and implementation. Currently, principles of evidence-based-medicine dominate almost all of these national guideline programs. The creation of international networks, like the GIN, as well as the conduct of projects like the AGREE, have clearly contributed to the improvement and standardization of these processes in the participant countries. Contrastingly, LMIC countries, with few exceptions, did not take part of these experiences. Diffusion and dissemination of appropiate methods for evidence-based guidelines development is limited in these countries. According to the results of this study, in Argentina, as late as 2004, this process was not systematized and still relied heavily on the opinion of experts.

Thirdly, standards proposed by the AGREE instrument could be relatively high for the context of a LMIC and specially if it is taken into account the fact that, except for the last two years (2003 and 2004), the period during which Argentine guidelines production was described preceded the year of diffusion of this instrument (2003). In some LMIC, language barriers and limited accesibility to updated biomedical literature can negatively impact on the use of relevant and important evidence to support guidelines recommendations. In this sense, an invaluable resource for Argentine guidelines developers is the Cochrane Library Plus, which can be freely accessed and contains the Spanish version of systematic reviews produced by the Cochrane Collaboration [21]. Even when currently there is broad agreement on the need for systematic reviews to inform recommendations, this type of evidence was rarely referred in Argentine guidelines [22]. Therefore, networking activities betweeen guideline producers and Argentine Cochrane Centers shoud also be promoted. As reported by Varonen and col., this kind of cooperation showed to be very positive in many senses [23].

Another factor that could have influenced quality of Argentine guidelines is the lack of economical and human resources devoted to guideline production. Since the cost of producing evidence-based guidelines is relatively too high for health budgets of LMIC countries, a systematic methodology to adapt international guidelines would be an efficient way of improving not only the quantity but also their quality [24]. Internationally developed guidelines can be adapted to the national context, representing a considerable saving of money. However, an explicit and systematic adaptation process should be performed as guidelines' applicability and transferability can be strongly influenced by different factors, e.g.: population needs (prevalence of disease, baseline risk status), setting (availability of resources) and other factors that modify translation of recommendations into practice [25,26]. In 2006, the National Academy of Medicine (NAM), in collaboration with the National Ministry of Health, developed and validated an adaptation process in order to increase the quality of guidelines produced in the country. Currently, a virtual learning course is implemented by NAM, with the purpose of improving national and local capacities in guideline adaptation [27].

Finally, findings of this assessment highlight the need of improving the reporting of the editorial independence of guideline producers. Practically none Argentine guideline reported conflict of interests or funding sources. Lack of transparency was also reported by Papanikolaou et al. in an evaluation of 191 published guidelines: only 7 (3.7%) disclosed potential conflicts of interest [28]. In the case of Argentine guidelines, omission could have been unintentional or, on the contrary, intentional (financial ties might have existed in some situations and deliberately hidden by guideline authors). However, regardless of the intent of guideline developers' actions, explicit declaration of conflict of interests at the begining of the process is strongly recommended by most international organizations as a way of reducing the probability of biased recommendations and increasing guidelines' credibility [29].

Some methodological issues must be addressed. First of all, evaluation was restricted to guidelines that were diffused and identified on Websites or in journals. Diffusion is not the same as development as there might have been guidelines produced and used in health institutions that could have not been identified by the searching strategies applied in this research. In spite of this limitation, the study was focalized on those guidelines diffused by well-known and reputed institutions in Argentina, which have a high probability of being adopted by healthcare professionals. Secondly, Internet searching was not exhaustive at the meso level, concretely in hospitals: as a reduced number of these institutions have Websites, only 3 out of 10 eligible hospitals could be assessed. Thirdly, even when the AGREE collaboration strongly suggests 4 appraisers per each CPG, this could not be performed because of lack of resources. All researchers and appraisers work ad-honorem. However, in spite of the inclusion of only two appraisers per guideline, reliability scores were acceptable. In fourth place, only CPG documents were considered for the assessment. Finally, there are some limitations inherent to the instrument applied. Quality of guideline is assessed on the basis of what is reported: quality of reporting is not the same as quality of the development process. As in other quality assessment studies, none content analysis of the recommendations was performed [30].

To our knowledge this is the first time a study of this kind has been undertaken in Argentina and Latin America, the Brazilian research excepted. Its execution was the first step in the building of a network of professionals interested in improving CPG development, dissemination and implementation in the country. Its findings might be very useful in the set up of a national evidence based guideline development program.

## Conclusion

This study was one of the firsts that systematically employed the AGREE instrument for the critical assessment of guidelines produced in a LMIC. The AGREE instrument can serve as a model to identify improvement opportunities in the guidelines development process of these countries. In this sense, this research shows the low quality of guidelines produced and points out areas to which training iniatiatives should be oriented.

Guideline development and diffusion in Argentina from 1994 to 2004 shows a constant increment, although quality of reporting did not improve; moreover, in some aspects it seemed to decline. Institutions involvement in this process was dispersed, rarely integrated, and not systematized. A national debate between main stakeholders is urgently needed in order to contribute to the definition of a clear and explicit policy for CPG development, dissemination and implementation in the country.

## Competing interests

The authors declare that they have no competing interests.

## Authors' contributions

MEE conceived the study, designed the protocol and coordination of the research, performed the Internet Search, registered pCPG in the database, selected the CPG from the database, was in charged of appraisers training and appraised guidelines, performed the statistical analysis, interpreted the data, drafted the manuscript. ZO participated in the design of the protocol and coordination of the research, selected the CPG from the database, interpreted the data and helped to draft the manuscript. MGD performed the database electronic searching, appraised guidelines, interpreted the data, and helped to draft the manuscript. ECh, RM and RB appraised guidelines, interpreted the data and helped to draft the manuscript. All the authors read and approved the final manuscript.
